# A Murine Monoclonal Antibody With Potent Neutralization Ability Against Human Adenovirus 7

**DOI:** 10.3389/fcimb.2019.00417

**Published:** 2019-12-04

**Authors:** Rong Wang, Jiansheng Lu, Quan Zhou, Lei Chen, Ying Huang, Yunzhou Yu, Zhixin Yang

**Affiliations:** Laboratory of Protein Engineering, Beijing Institute of Biotechnology, Beijing, China

**Keywords:** human adenovirus type 7 (HAdV-7), mouse monoclonal antibody (MMAb), immune library, neutralizing antibody, antiviral drugs

## Abstract

B1-type human adenoviruses (HAdVs) HAdV-3, HAdV-7, and HAdV-55 have caused epidemics in North America, Asia, and Europe. However, to date, no adenovirus vaccines or antiviral drugs have been approved for general use. In the present work, a scFv-phage immune library was constructed and mouse monoclonal antibody (MMAb) 10G12 was obtained through selection. 10G12 is specific for HAdV-7 and binds the hexon loop1 and loop2 (LP12), resulting in strong neutralization activity against HAdV-7. Additionally, it is stable in serum and buffer at various pH values. The findings provide insight into adenovirus and antibody responses and may facilitate the design and development of adenovirus vaccines and antiviral drugs.

## Introduction

Human adenoviruses (HAdVs), non-enveloped, icosahedral, double-stranded DNA viruses spanning > 85 genotypes, are classified into seven species (A–G) (Yoshitomi et al., 2017). HAdV infection is characterized by a broad spectrum of disease symptoms in humans, including sore throat, pneumonia, fever, and acute otitis media, with most cases involving gastrointestinal symptoms that vary with infection genotype (Arnold et al., 2010; Kunz and Ottolini, 2010). Symptoms are generally mild and self-limiting in immune-competent adults, but outbreaks of acute respiratory diseases (ARDs), such as community-acquired pneumonia (CAP), can occur in newborns, school students, and military recruits (Tan et al., 2016). B1 type adenoviruses HAdV-3, HAdV-7, and HAdV-55 are responsible for most epidemics in North America, Asia, and Europe (Choi et al., 2005; Zhang et al., 2006; James et al., 2007; Selvaraju et al., 2011; Tang et al., 2011; Gopalkrishna et al., 2016). To date, no vaccines for the general population available for HAdVs, and only vaccines against HAdV types 4 and 7 have been developed for the USA military (Russell et al., 2006; Kajon et al., 2015). Additionally, no antiviral drugs or efficient antiviral therapies have been approved for treating HAdVs (Echavarría, 2008).

Immune reconstitution plays a critical role in controlling AdV infection, and serotype-specific neutralizing monoclonal antibodies (MAbs) correlate with clearance of AdV (Heemskerk et al., 2005; Echavarría, 2008). The adenovirus capsid is formed from three major proteins (hexon, penton base, and fiber) and four minor proteins (IIIa, VI, VIII, and IX). Hexon, the most abundant capsid protein, recruits cytoplasmic dynein, a crucial component for transporting viral capsids along microtubules (Scherer and Vallee, 2015). Hexon is also an important antigen for neutralizing antibodies against HAdV-3, -5, -7, -14, and -55 (Sumida et al., 2005; Tian et al., 2011; Yu et al., 2013; Su et al., 2016). Type-specific neutralization epitopes on hexon proteins of many adenoviruses are primarily located in seven hypervariable regions (Rux et al., 2003; Pichla-Gollon et al., 2007; Yuan et al., 2009; Bradley et al., 2012; Qiu et al., 2012; Tian et al., 2018b). Hexon stimulates type-specific neutralizing Abs (NAbs), whereas fiber induces Abs with cross-neutralizing activity against HAdV-14 and HAdV-55 (Feng et al., 2018). However, to date, only a few neutralization epitopes on the HAdV fiber knob region have been identified.

In the present study, scFv 10G12 was screened from an scFv-phage antibody immune library and subcloned to generate an MMAb. We identified MMAb 10G12 as a potent antibody that effectively targets HAdV-7 *in vitro* at low concentrations by binding to hexon loop1 and loop2 (LP12). MMAb 10G12 displayed good stability in serum and phosphate buffer (PB) at different pH values.

## Materials and Methods

### Cell Lines and Viruses

HEK293F and A549 cells (ATCC, USA) were cultured in Dulbecco's modified Eagle's medium (DMEM) containing 10% fetal bovine serum (FBS) (Excell, China). FreeStyle™ 293-F cells (Invitrogen, USA) were cultured in FreeStyle^TM^ 293 Expression Medium (12338; Gibco, USA). Cells were incubated at 37°C in a 5% CO_2_ atmosphere. The HAdV-7 GZ6965 strain (human/CHN/GZ6965/2001) used herein was obtained as described previously (Qiu et al., 2012) and maintained in our laboratory. The HAdV-55 strain was isolated from a patient and kindly provided by Prof. Hongbin Song (Center for Disease Control and Prevention of Chinese PLA, Beijing, China). HAdV-7 and HAdV-55 were propagated in HEK293-F cells grown in DMEM containing 2% FBS. When 75–95% of cells exhibited typical cytopathic effects (CPEs) consistent with HAdV infection, the cell suspension was frozen at −80°C and thawed three times, centrifuged at 4,000 g for 5 min, and the supernatant was inactivated and purified using standard CsCl gradient centrifugation (Wu et al., 2002). The obtained virus particles were aliquoted and stored at −80°C.

### Construction and Selection of scFv-phage Antibody Immune Libraries

Preparation and characterization of the scFv-phage display library was subsequently performed. Female BALB/c mice at 6–8 weeks old were immunized with inactivated HAdV-7. Pre-immune sera were collected from mouse tails and used as negative controls. A 100 μg sample of inactivated HAdV-7 emulsified in Freund's complete adjuvant (Sigma, USA) was intraperitoneally injected, followed by four boosters of the same dose at 2-week intervals. Spleens were harvested 3 days after the final booster, and total RNA was isolated from spleen cells and was reverse transcribed into cDNA (K1621, Thermo Scientific, USA). Primers for reverse-transcription were PmCGR (TGCATTTGAACTCCTTGCC) and PmCKR (CCATCAATCTTCCACTTGAC). Full-length variable light (V_L_) and variable heavy (V_H_) chain genes were amplified by overlay-extended PCR and the scFv fragment was cloned into phage display vector pADSCFV-S. Competent *Escherichia coli* HST08 Blue cells were transformed with the ligation mixture by electroporation. Transformed cells were titrated on agar plates to determine the library size, and colony PCR was performed on a selection of colonies to determine the presence of DNA inserts in the vector. Harvested cells samples harboring the final scFv antibody gene library were combined, aliquoted, and stored at −80°C.

Purified HAdV-7 (300 ng, 100 μl) in PBS was incubated in a microtiter plate well overnight at 4°C, then blocked with 3% BSA in TBS (50 mM Tris-HCl pH 7.5, 150 mM NaCl) for 2 h at 37°C. A 100 μl sample of phage library at 1.9 × 10^7^ plaque-forming units (pfu) per ml was added and incubated for an additional 2 h at 37°C after a washing step. After washing, wells with TBST (TBS containing 0.05% Tween-20), bound phage was eluted with 120 μl 0.1 M glycine-HCl (pH 2.2) and neutralized with 15 μl 1 M Tris-HCl (pH 9.0). After eluting, phage was amplified by infecting *E. coli* XL1-Blue cells, and four rounds of panning were carried out. Positively selected phages were amplified and resulting scFv was subjected to DNA sequence.

### MMAb Generation of scFv

PCR was performed to amplify the full-length variable light (V_L_) and variable heavy (V_H_) chain genes of positively selected phages. PCR products were digested with restriction endonucleases *Sal* I and *Age* I, then cloned separately into pMABG1 and pMABKa vectors containing a mouse immunoglobulin constant gene. Recombinant antibodies were obtained as IgG1 molecules, regardless of their original isotype. FreeStyle 293-F cells were transfected with equal quantities of plasmids encoding heavy and light chains using a FectoPRO transfection kit (116-001, Polyplus-Transfection, French) for antibody expression. At 4 days after transfection, antibody-containing supernatants were harvested, and antibodies were purified using HiTrap MabSelect Xtra (28-4082-60, GE Healthcare, USA).

### Expression and Purification of Loop1 and Loop2 (LP12) and Fiber

Viral DNA was extracted from A549 cells infected with HAdV-7 or HAdV-55 using DNAVzol (Vigorous, Beijing, China) following the manufacturer's instructions. Genes encoding the hexon LP12 fragment and fiber were amplified by PCR and inserted into the pTIG-TRX vector. Primers used for PCR are listed in Table 1 (7LP12, LP12 of HAdV-7; 55LP12, LP12 of HAdV-55; 7Fiber, Fiber of HAdV-7; 55Fiber, Fiber of HAdV-55). The pTIG-TRX-LP12 plasmid was transformed into *E. coli* BL21 (DE3) cells (TransGen, Beijing, China) for expression of His-tagged fusion protein. Transformed cells were cultured in Luria-Bertani medium containing ampicillin at 37°C, and recombinant expression was induced with 0.6 mM isopropyl b-D-thiogalactoside (IPTG) when the absorbance at 600 nm (OD600) reached 0.4–0.6. After reducing the temperature from 37°C to 16°C, cells were cultured for a further 16 h. Bacteria were lysed by ultrasonic treatment, and recombinant protein was purified by Ni-agarose resin.

**Table 1:** Sequences of primers used for PCR.

**Primer name**	**Primer sequences (5^**′**^-3^**′**^)**
7LP12-F	AGGAATTCTAATGGGATCCCACCACCATCATCATCATACATCTCAGTGGATAGTTACA
7LP12-R	GTGCTCGAGCTCGAGCTATTAATTGTCCATTGGGTCAAG
55LP12-F	AGGAATTCTAATGGGATCCCACCACCATCATCATCATAGTTTCAAACCCTATTCTGGTAC
55LP12-R	GTGCTCGAGCTCGAGCTATTACCGCCCGTTCATGTAGTCGTA
7Fiber-F	AGGAATTCTAATGGGATCCCACCACCATCATCATCATACCAAGAGAGTCCGGCTCA
7Fiber-R	GGTGCTCGAGTCATTAGTCGTCTTCTCTGATGTA
55Fiber-F	AGGAATTCTAATGGGATCCCACCACCATCATCATCATACCAAGAGAGTCCGGCTCAGT
55Fiber-R	GGTGCTCGAGTCATTAGTCGTCTTCTCTGATGTAG

### ELISA Assay

Wells of ELISA assay plates (9018, Costar, USA) were coated with 200 ng antigen and incubated overnight at 4°C. Wells were then blocked with 200 μl of 5% (w/v) skimmed milk-PBS for 2 h at 37°C, and 200 μl of antibody was added and incubated for 2 h at 37°C. Plates were washed three times with PBS-Tween (0.1% v/v), and goat anti-mouse horseradish peroxidase (HRP)-conjugated IgG antibody (1:5,000, v/v) was added and incubated for 1 h at 37°C. Finally, three rounds of washing with PBS-Tween were carried out, and detection at 492 and 630 nm was performed using OPD chromogen substrate.

To examine whether MMAb 10G12 was specific for HAdV-7, inactivated HAdV-7 and HAdV-55 were used as antigens, and inactivated influenza virus H3N2 (A/swine/Colorado/1/77) (Karasin et al., 2000) and a synthetic polypeptide antigen of foot-and-mouth disease virus (FMDV) (ETQVQRRQHTDVSFILDRFVKVTPKDQINALDLMQTPAHTEPGSRVTNVRGDLQVLAQKAARTLPPGSRHKQKIVAPVKQLL) served as negative controls. The MMAb antibody 10G12 was used at a concentration of 7.5 μg/mL. To examine the affinity of MMAb 10G12 for HAdV-7, inactivated HAdV-7 was used as antigen, and the MMAb 10G12 antibody was 2-fold serially diluted from an initial concentration of 80 μg/mL to 19.07 pg/mL. To identify which epitope MMAb 10G12 binds, fiber, and the hexon LP12 from HAdV-7 and HAdV-55 were used as antigens, and MMAb 10G12 antibody employed at 7.5 μg/mL.

### Virus Neutralization Test

For *in vitro* adenovirus neutralization experiments, 100 μl of A549 cells (3 × 10^5^ cells/ml) were seeded in each well of 96-well plates incubated overnight at 37°C in a 5% CO_2_ atmosphere. Purified 10G12 was serially diluted 2-fold from 25 to 0.1 μg/ml in DMEM, and 50 μl aliquots of each dilution were mixed with 50 μl HAdV-7 or HAdV-55 with 100TCID_50_. Anti-DENV1 (Lu et al., 2018) and anti-EGFR (CN102993305B) antibodies served as negative controls. Antibody-virus mixtures were incubated at 37°C for 1 h, transferred to 96-well plates containing 85–95% confluent A549 cell monolayers, and cultured in DMEM without Phenol Red or serum for 72 h. Infected cells were observed under a microscope and the number of holes in cells with lesions was counted.

To test the ability of MMAb to rescue HAdVs infection, 100 μl of A549 cells (3 × 10^5^ cells/ml) were seeded in each well of 96-well plates and incubated overnight at 37°C in a 5% CO_2_ atmosphere. Next, 100 μl samples of HAdV-7 or HAdV-55 with 100TCID_50_ were added to 96-well plates containing 85–95% confluent A549 cell monolayers and incubated at 37°C for 1 h. Purified 10G12 was serially diluted 2-fold from 25 to 0.1 μg/ml in DMEM without Phenol Red and serum, and 100 μl aliquots of each dilution were added to 96-well plates and incubated for 1 week at 37°C. Anti-DENV1 and anti-EGFR antibodies served as negative controls. Infected cells were observed under a microscope and the number of holes in cells with lesions was counted.

### MMAb Stability Analysis

To test the stability of MMAb in serum, purified 10G12 was diluted in fetal bovine serum (FBS, Excell) to 25 μg/ml and incubated for 3, 7, or 10 days at 37°C. ELISA assays were then performed to detect whether samples still efficiently recognized HAdV-7.

To test the MMAb stability in PB at different pH values, purified 10G12 was diluted in PB at pH 6.0, 6.5, 7.0, 7.5, and 8.0, and incubated at 37°C for 5 or 8 days. ELISA assays were then performed to detect whether samples still efficiently recognized HAdV-7.

### Western Blotting Assay

Purified antigens were quantified using a NanoDrop One^c^ instrument (Thermo, USA). A 7.5 μg sample of reduced protein was separated by SDS-PAGE and subsequently transferred to a polyvinylidene fluoride (PVDF) membrane. Primary antibody 10G12 (1 mg/mL) was diluted 1:1,000, and secondary antibody HRP-goat anti-mouse immunoglobulin G (IgG) (ZSGB-BIO) was diluted 1:5,000. Signals were detected using Western HPR Substrate Peroxide solution (Millipore).

### Statistical Analysis

All experiments were repeated at least three times, except for the stability assay. Data are presented as means ± standard deviation (SD). Statistical significance was determined using GraphPad Prism 5.0 software. An affinity graph was plotted, and EC_50_ values were determined using GraphPad Prism 5.0 software, too. The significance of differences in protective effects compared with controls was evaluated using two-tailed Student's *t*-tests, and *p*-values < 0.05 were considered statistically significant.

## Results

### Construction and Selection of scFv-phage Antibody Immune Libraries

Female BALB/c mice at 6–8 weeks old were immunized with inactivated HAdV-7, and spleens were harvested for RNA extraction after four booster injections. Genes encoding V_L_ and V_H_ chains were amplified by PCR, and DNA fragments of the expected size (350 bp) were obtained. Overlay-extended PCR was performed to generate scFv DNA fragments of ~750 bp, which were then cloned into the phage vector pADSCFV-S. The final scFv antibody gene library consisted of 1.9 × 10^7^ independent clones, with 80% correctness.

**Figure 1:**
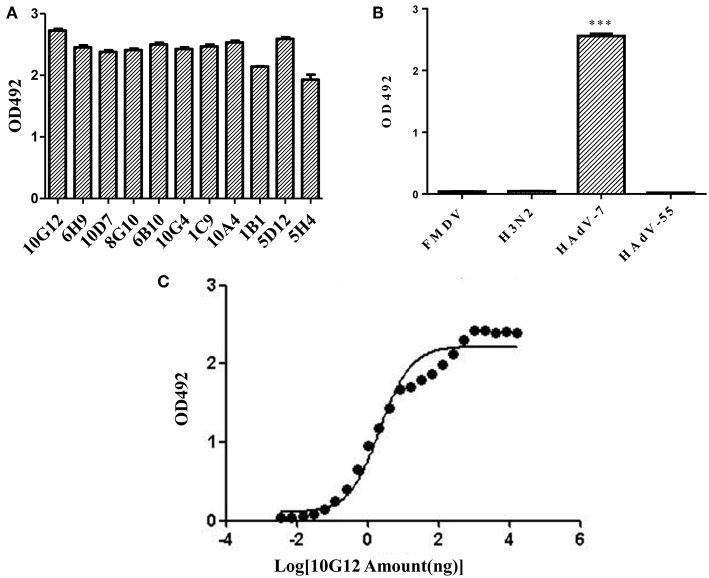
Identification of mouse monoclonal antibodies (MMAbs) against HAdV-7. **(A)** Screening of scFv-displaying phage by ELISA. After three round of panning, 11 positive clones were identified that recognized HAdV-7. **(B)** ELISA analysis of binding between MMAb 10G12 and various antigens. The synthetic polypeptide antigen of foot-and-mouth disease virus (FMDV) and influenza A virus H3N2 (A/swine/Colorado/1/77) served as negative controls. HAdV-55 was tested for potential cross-reactivity. Results are presented as means ± SD from three independent experiments (***p < 0.001 vs. negative controls calculated by *t*-tests). **(C)** Affinity curve of the ELISA results for binding between HAdV-7 and serially diluted MMAb 10G12. ELISA assays were performed with 200 ng of inactivated HAdV-7 per well. MMAb 10G12 was serially diluted from an initial concentration of 80 μg/mL.

In total, 11 positive clones were identified from samples after the third round of panning against HAdV-7, and their ability to interact with HAdV-7 is shown in Figure 1A. These phagemids were extracted and each insert was sequenced. The results revealed five unique full-size scFv sequences among the 11 clones (10G12, 6H9, 10D7, 8G10, 6B10, 10G4, and 1C9 shared the same sequence; 5H4 had the wrong sequence). V_H_ and V_L_ of 10G12, 10A4, 1B1, and 5D12 were recloned into pMABG1 or pMABKa to generate murine IgG1 molecules. Although these four antibodies were specific to HAdV-7, 10A4, 1B1, and 5D12 did not exhibit neutralizing activity (data not shown). Thus, subsequent experiments only characterized 10G12.

### MMAb 10G12 Is Specific for HAdV-7

To examine whether MMAb 10G12 is specific for HAdV-7, an ELISA assay was performed. Since positive clones bound to various unrelated viruses in the previous selection of the scFv-phage antibody library, two unrelated antigens (influenza virus H3N2 and the synthetic polypeptide antigen of FMDV) were included as negative controls. Additionally, since there are more than 85 HAdV genotypes, HAdV-55 was tested for potential cross-reactivity with MMAb 10G12. As shown in Figure 1B, MMAb 10G12 bound to inactivated HAdV-7, but not to inactivated influenza virus H3N2, the synthetic polypeptide antigen of FMDV, or HAdV-55. Furthermore, ELISA assay was performed to test the affinity of MMAb 10G12 for HAdV-7. Based on the absorbance at 492 nm, and affinity graph was plotted using GraphPad Prism 5.0 software (Figure 1C). The resulting EC_50_ values indicated that the affinity between MMAb and HAdV-7 was 0.14 nM.

### *In vitro* Neutralizing Activity and Therapeutic Effects of MMAb 10G12

To examine the neutralization potential of MMAb 10G12, *in vitro* adenovirus neutralization experiments were performed using A549 cells. Purified 10G12 was serially diluted 2-fold from 25 to 0.1 μg/ml in DMEM, and 50 μl aliquots of each dilution were mixed with 50 μl HAdV-7 or HAdV-55 with 100TCID_50_. The antibody-virus mixtures were transferred to A549 cells, and every dilution included eight replicates. After 72 h, infected cells were observed under the microscope, and wells containing surviving cells were counted. Two antibodies (anti-DENV and anti-EGFR) served as negative controls. As shown in Table 2, 50 μl aliquots of 10G12 with 0.4 μg/ml could neutralize 100% of 50 μl HAdV-7 with 100TCID_50_, and all cells at this dilution had no lesions. Even 50 μl aliquots of 10G12 with 0.2 μg/ml could neutralize 50% of 50 μl HAdV-7 with 100TCID_50_, but cells at this dilution had partial lesions. Furthermore, even 10G12 at 25 μg/ml was unable to neutralize HAdV-55 with 100TCID_50._ These findings indicate that MMAb 10G12 exhibited strong neutralization activity against HAdV-7 and poor cross-reactivity with HAdV-55.

**Table 2:** *In vitro* neutralization activity of MMAb 10G12 (surviving cell holes/total).

**Virus**	**Antibody**	**Antibody concentration (μg/ml)**								
		**25**	**12.5**	**6.25**	**3.2**	**1.6**	**0.8**	**0.4**	**0.2**	**0.1**
No	10G12	8/8	8/8	8/8	8/8	8/8	8/8	8/8	8/8	8/8
HAdV-7	Anti-DENV1	2/8	0/8	0/8	0/8	0/8	0/8	0/8	0/8	0/8
HAdV-7	Anti-EGFR	0/8	0/8	0/8	0/8	0/8	0/8	0/8	0/8	0/8
HAdV-7	No	0/8	0/8	0/8	0/8	0/8	0/8	0/8	0/8	0/8
HAdV-7	10G12	8/8	8/8	8/8	8/8	8/8	8/8	8/8	4/8	0/8
HAdV-55	10G12	0/8	0/8	0/8	0/8	0/8	0/8	0/8	0/8	0/8

Next, the ability of MMAb 10G12 to rescue HAdVs infection was investigated to explore potential therapeutic effects. HAdV-7 or HAdV-55 with 100TCID_50_ were added to 96-well plates containing 85–95% confluent monolayers of A549 cells and incubated at 37°C for 1 h. Purified 10G12 was then serially diluted 2-fold from 25 to 0.1 μg/ml, added to infected A549 cells and incubated for 1 week at 37°C. Anti-DENV1 and anti-EGFR antibodies served as negative controls. Infected cells were observed under a microscope and the number of holes in cells with lesions was counted. As shown in Table 3, even at 1 h after infection, 3.2 μg/ml 10G12 could rescue 100% of cells infected with 100TCID_50_ HAdV-7, and none of the cells at this dilution displayed lesions. Even 0.8 μg/ml 10G12 could protect 25% of cells infected with 100TCID_50_ HAdV-7. Furthermore, even 25 μg/ml 10G12 was unable to rescue A549 cells infected with 100TCID_50_ HAdV-55. These findings indicate that MMAb 10G12 exhibited potent therapeutic effects against HAdV-7.

**Table 3:** *In vitro* therapeutic effects of MMAb 10G12 (surviving cell holes/total).

**Virus**	**Antibody**	**Antibody concentration (μg/ml)**								
		**25**	**12.5**	**6.25**	**3.2**	**1.6**	**0.8**	**0.4**	**0.2**	**0.1**
No	10G12	8/8	8/8	8/8	8/8	8/8	8/8	8/8	8/8	8/8
HAdV-7	Anti-DENV1	0/8	0/8	0/8	0/8	0/8	0/8	0/8	0/8	0/8
HAdV-7	Anti-EGFR	0/8	0/8	0/8	0/8	0/8	0/8	0/8	0/8	0/8
HAdV-7	No	0/8	0/8	0/8	0/8	0/8	0/8	0/8	0/8	0/8
HAdV-7	10G12	8/8	8/8	8/8	8/8	6/8	2/8	0/8	0/8	0/8
HAdV-55	10G12	0/8	0/8	0/8	0/8	0/8	0/8	0/8	0/8	0/8

### MMAb 10G12 Is Stable in Serum and PB at Different pH Values

**Figure 2:**
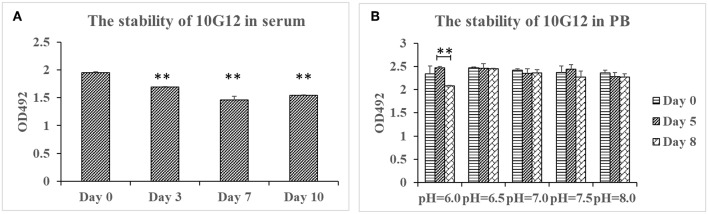
Stability of 10G12 in serum and PB at different pH values. **(A)** ELISA analysis of the binding between HAdV-7 and MMAb 10G12 in serum. Purified 10G12 was diluted in fetal bovine serum to 25 μg/ml and incubated at 37°C for 3, 7, and 10 days. ELISA assays were performed with 200 ng of inactivated HAdV-7 per well. **(B)** ELISA analysis of binding between HAdV-7 and MMAb 10G12 in PB at different pH values. Purified 10G12 was diluted in PB at pH 6.0, 6.5, 7.0, 7.5, and 8.0 and incubated at 37°C for day 5 and 8. ELISA assays were performed with 200 ng inactivated HAdV-7 per well. Data were obtained from two separate experiments, and results are presented as means ± SD (**p < 0.01 vs. day 0 values calculated by *t*-test).

To test MMAb stability in serum, purified 10G12 was diluted in FBS to 25 μg/ml and incubated in 37°C for 3, 7, and 10 days. ELISA assays were then performed to detect whether samples still efficiently recognized HAdV-7. As shown in Figure 2A, compared with day 0, the binding activity of samples diluted in FBS after 3, 7, and 10 days decreased by a statistically significant amount. However, even after 10 days of incubation at 37°C, the binding activity was only decreased 20%. Additionally, from day 3 to 10, the binding activity did not decrease with increasing incubation duration. Differences between fetal bovine serum and the mouse monoclonal antibody may explain this decrease in binding activity. The results indicate that MMAb 10G12 was relatively stable in serum.

To test the MMAb stability in PB at different pH values, purified 10G12 was diluted in PB at pH 6.0, 6.5, 7.0, 7.5, and 8.0, and incubated at 37°C for 5 or 8 days. ELISA assays were then performed to detect whether samples still efficiently recognized HAdV-7. As shown in Figure 2B, samples diluted in PB at pH 6.0, 6.5, 7.0, 7.5, and 8.0 after 8 days of incubation still had the same binding activity as those before incubation. These results indicate that MMAb 10G12 is stable in serum and PB at different pH values.

### MMAb 10G12 Binds Hexon Loop1 and Loop2

**Figure 3:**
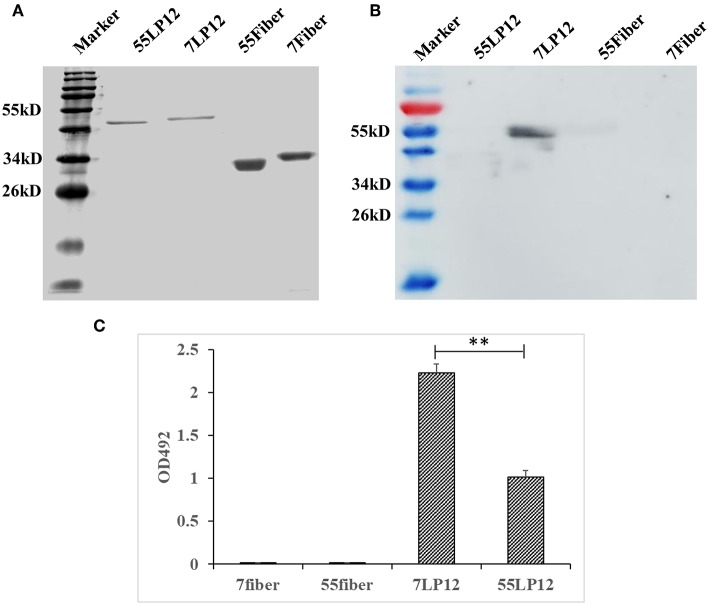
Binding of MMAb 10G12 to loops 1 and 2 (LP12) of hexon. **(A)** Reducing polyacrylamide gel electrophoresis analysis of purified antigens. A 5 μg sample of reduced Fiber and LP12 of HAdV-7 and HAdV-55 was separated by SDS-PAGE and subsequently stained with Coomassie Brilliant Blue. **(B)** Western blotting analysis of binding between 10G12 and various antigens. A 7.5 μg sample of reduced Fiber and LP12 of HAdV-7 and HAdV-55 was separated by SDS-PAGE and subsequently transferred to a PVDF membrane. Primary antibody 10G12 (1 mg/mL) was diluted 1:1,000, and secondary antibody HRP-goat anti-mouse IgG was diluted 1:5,000. Signals were detected using Western HPR Substrate Peroxide solution. **(C)** ELISA analysis of binding between 10G12 and various antigens. Data were obtained from three separate experiments, and results are presented as means ± SD (**p < 0.05 between 7LP12 and 55LP12, calculated by *t*-tests).

Hexon is an important antigen of neutralizing antibodies, and fiber also has a few neutralization epitopes. To determine to which epitope MMAb 10G12 binds, fragments comprising loop1 and loop2 (LP12) of hexon and fiber from HAdV-7 and HAdV-55 were amplified by PCR and subcloned into pTIG-TRX. Recombinant proteins were expressed and purified (Figure 3A), and the results of western blotting showed that MMAb 10G12 bound LP12 of HAdV-7 but not HAdV-55 or fiber (Figure 3B). When using reduced protein samples for SDS-PAGE, 10G12 should bind the linear epitopes of 7LP12. Thus, ELISA assay plates were coated with LP12 or fiber from HAdV-7 and HAdV-55 at 200 ng per well, and the affinity for MMAb 10G12 was measured. As shown in Figure 3C, MMAb 10G12 bound LP12 of HAdV-7 but not fiber, consistent with the results of western blotting in Figure 3B. However, the ELISA result showed that 10G12 bound LP12 of HAdV-55 with weaker affinity than HAdV-7. Combined with the western blotting results in Figure 3B, this indicates that 10G12 might bind some spatial epitopes of 55LP12. However, 10G12 did not neutralize HAdV-55 (Table 2), suggesting that these might be non-neutralizing spatial epitopes. In summary, MMAb 10G12 clearly bound the hexon LP12 region.

## Discussion

In this study, we identified antibody MMAb 10G12 that binds specifically to HAdV-7 through the hexon LP12 region. MMAb 10G12 exhibited strong neutralization activity against HAdV-7 and was stable in serum and PB at different pH values.

Despite more than 85 genotypes have been identified for HAdVs, few neutralizing antibodies have been reported. Tian et al. (2018a) reported that the recombinant trimeric HAdV-11 fiber knob region is responsible for cross-neutralizing antibody responses against HAdV-11, -7, -14p1, and -55 in mice. Three neutralizing MAbs, 6A7, 3F11, and 3D8, were obtained via mouse hybridoma fusion, of which 3F11 and 3D8 cross-neutralized HAdV-11,-7, and -55, but not HAdV-14p1 (Tian et al., 2018a). Feng et al. (2018) previously demonstrated that a fiber-specific antibody in sera contributed to cross-neutralizing activity against HAdV-14 and HAdV-55. However, HAdV-55 is a recombinant chimera of HAdV-11 and -14 that contains the fiber gene from HAdV-14. Our current study shows that 10G12 does not neutralize HAdV-55, and has poor cross-reactivity, probably through interaction with non-neutralizing epitopes.

MMAb 10G12 is a murine neutralizing antibody like 6A7, 3F11, and 3D8 (Tian et al., 2018a). Monoclonal antibodies of mouse origin can induce human anti-mouse antibody (HAMA) responses, which restricts the use of MMAb in humans (Hertel et al., 1990). The first MMAb, Muromonab, has been on the market since 1992, and humanized antibodies based on murine MAb are increasingly being developed (Makulska-Nowak, 1993). In future work, MMAb 10G12 may be further humanized for therapeutic use.

In this study, we expressed and purified the recombinant LP12 hexon region. ELISA assay plates were coated with 200 ng of inactivated HAdV-7 or LP12 per well, and the affinity for MMAb remained the same (Figures 1B, 3C). This indicates that LP12 is present in the main neutralization epitopes of HAdV-7. Previous studies confirmed that hexon protein, the most abundant capsid protein, is the predominant target of neutralizing antibodies (NAbs) recognizing HAdV-3, -5, -7, -14, and -55 (Sumida et al., 2005; Tian et al., 2011; Bradley et al., 2012; Yu et al., 2013; Su et al., 2016; Feng et al., 2018). Additionally, the affinity results indicate that LP12 could substitute for inactivated HAdV-7 in initial screening and testing.

In summary, MMAb 10G12 was specific for HAdV-7 and displayed good stability. In future work, we will explore whether MMAb 10G12 can provide protection against HAdV-7 *in vivo*, and if so, MMAb may be further humanized for use as a therapeutic agent.

## Data Availability Statement

The data used to support the findings of this study are available from the corresponding author upon request.

## Ethics Statement

The animal study was reviewed and approved by Academy of Military Medical Sciences (AMMS; ID: SYXK2012–05).

## Author Contributions

JL and ZY conceived this study. YH, YY, and ZY carried out experiments. RW, YH, LC, and QZ performed data analysis. RW and ZY drafted, wrote, edited, and reviewed the manuscript. ZY acquired funding. JL and QZ provided resources. RW, YH, YY and ZY supervised the work.

### Conflict of Interest

The authors declare that the research was conducted in the absence of any commercial or financial relationships that could be construed as a potential conflict of interest.
